# The prevalence and clinical manifestation of hereditary thrombophilia in Korean patients with unprovoked venous thromboembolisms

**DOI:** 10.1371/journal.pone.0185785

**Published:** 2017-10-17

**Authors:** Su Yeon Lee, Eun Kyoug Kim, Min Sun Kim, Sun Hye Shin, Haseong Chang, Shin Yi Jang, Hee-Jin Kim, Duk-Kyung Kim

**Affiliations:** 1 Division of Cardiology, Department of Medicine, Samsung Medical Center, Sungkyunkwan University School of Medicine, Seoul, Republic of Korea; 2 Division of Cardiology, Heart Vascular Stroke Institute, Samsung Medical Center, Sungkyunkwan University School of Medicine, Seoul, Republic of Korea; 3 Division of Pulmonary and Critical Care Medicine, Department of Medicine, Samsung Medical Center, Sungkyunkwan University School of Medicine, Seoul, Republic of Korea; 4 Department of Laboratory Medicine & Genetics, Samsung Medical Center, Sungkyunkwan University School of Medicine, Seoul, Republic of Korea; National Cerebral and Cardiovascular Center, JAPAN

## Abstract

**Background:**

Hereditary thrombophilia (HT) is a genetic predisposition to thrombosis. Asian mutation spectrum of HT is different from Western ones. We investigated the incidence and clinical characteristics of HT in Korean patients with unprovoked venous thromboembolism (VTE).

**Methods:**

Among 369 consecutive patients with thromboembolic event who underwent thrombophilia tests, we enrolled 222 patients diagnosed with unprovoked VTE. The presence of HT was confirmed by DNA sequencing of the genes that cause deficits in natural anticoagulants (NAs). Median follow-up duration was 40±38 months.

**Results:**

Among the 222 patients with unprovoked VTE, 66 (29.7%) demonstrated decreased NA level, and 33 (14.9%) were finally confirmed to have HT in a genetic molecular test. Antithrombin III deficiency (6.3%) was most frequently detected, followed by protein C deficiency (5.4%), protein S deficiency (1.8%), and dysplasminogenemia (1.4%). The HT group was significantly younger (37 [32–50] vs. 52 [43–65] years; P < 0.001) and had a higher proportion of male (69.7% vs. 47%; P = 0.013), more previous VTE events (57.6% vs. 31.7%; P = 0.004), and a greater family history of VTE (43.8% vs. 1.9%; P < 0.001) than the non-HT group. Age <45 years and a family history of VTE were independent predictors for unprovoked VTE with HT (odds ratio, 9.435 [2.45–36.35]; P = 0.001 and 92.667 [14.95–574.29]; P < 0.001).

**Conclusions:**

About 15% of patients with unprovoked VTE had HT. A positive family history of VTE and age <45 years were independent predictors for unprovoked VTE caused by HT.

## Introduction

Venous thromboembolism (VTE) is increasingly recognized as a significant source of morbidity and mortality [1]. It occurs in about one in 1000 people each year in Western countries; however, in Asian populations, VTE incidence is lower [2–4]. Although various factors such as immobilization, surgery, pregnancy, and malignancies increase the risk of VTE, in 25 to 50 percent of cases, no predisposing factor is present, and that is called *unprovoked VTE* [1,5,6].

Compared with patients with provoked VTE, those with unprovoked VTE tend to have higher recurrence rate, which means they must be treated for a long time. One of the main causes of this clinical difference is a genetic variant, hereditary thrombophilia (HT), associated with a predisposition to thrombosis [7,8]. The mutation spectrum of HT and the subsequent clinical manifestations vary among ethnic groups. Activated protein C (APC) resistance, caused by factor V Leiden mutation and prothrombin G20210A mutation, increases pro-coagulant activity and occurs only in Western populations [9–11]. A deficiency of natural anticoagulants (NAs), including antithrombin (AT), protein C (PC), protein S (PS), and plasminogen, is more likely to occur in Asian populations [11–13]. However, few data are available on the prevalence and clinical characteristics of Asian HT patients with unprovoked VTE. Furthermore, previous studies mostly defined HT not as a genetic abnormality, but as a deficiency of coagulation factors, which caused varied prevalence results [14–18]. Therefore, we aimed to identify the actual incidence and clinical manifestations of HT confirmed by genetic testing in Korean patients diagnosed with unprovoked VTE.

## Methods

### Study design and patients

We reviewed 369 consecutive patients with thromboembolic event who underwent coagulation testing at our VTE clinic between February 2005 and December 2015. The diagnosis of VTE was based on multi-detectable computed tomography (CT) scans and duplex sonography of the suspicious site. VTE included pulmonary embolism (PE), chronic thromboembolic pulmonary hypertension, deep vein thrombosis (DVT) of extremities, portal vein thrombosis, splanchnic vein thrombosis, and cerebral vein thrombosis. Among those patients, we included only adults (≥ 20 years old) confirmed to have unprovoked VTE, defined as a thrombotic event that occurred in the absence of predisposing factors such as immobilization for more than three days, surgery under anesthesia lasting for more than 30 minutes, pregnancy, connective tissue disease, malignancies and thrombogenic condition which was central venous line or pacemaker. We excluded patients diagnosed with arterial thromboembolism and those with underlying liver disease or acquired thrombophilia (e.g., antiphospholipid syndrome, *JAK2* mutation, or myeloproliferative neoplasm) [1,5,6].

We recorded each patient’s previous history of VTE, family members who experienced VTE, site of thromboembolism, initially presented symptoms, and comorbidities (diabetes, hypertension, and obesity). Hypertension was defined as systolic blood pressure > 140 mmHg or self-reported hypertension irrespective of pharmacologic treatment. Diabetes mellitus was defined as a history of type 1 or type 2 diabetes mellitus treated either pharmacologically or by diet. Obesity was defined as a body mass index greater than 25 kg/m^2^ based on Asian criteria. During the follow-up period, we investigated the duration of anticoagulation therapy and recurrence of VTE at any location. The protocol of this study was approved by the Institutional Review Board of the Samsung Medical Center, Seoul, Korea.

### Coagulation tests for thrombophilia

The coagulation tests used to screen for HT were PC activity (Stachrom^®^ Protein C, Diagnostica Stago, Asnières, France), PS free Ag (Liatest^®^ Free Protein S, Diagnostica Stago), AT activity (Stachrom^®^ AT III, Diagnostica Stago), and plasminogen activity (Stachrom^®^ Plasminogen, Diagnostica Stago), per the international guidelines [19]. All coagulation tests were performed on the STA^®^-Evolution Coagulation Analyzer. Reference ranges were determined according to our institutional data. Whenever possible, coagulation tests were repeated 2 weeks after the discontinuation of anticoagulation therapy if results at the time of diagnosis showed low levels of multiple NAs or if tests with abnormal results were performed under anticoagulant use.

### Molecular genetic tests

Patients who had low levels of NAs underwent molecular genetic tests to confirm HT. Genomic DNA was extracted from peripheral blood leukocytes using the Wizard Genomic DNA Purification kit (Promega, Madison, WI, USA). All exons of the *PROC*, *PROS1*, *SERPINC1*, and *PLG* genes and their flanking intron regions were amplified using polymerase chain reaction for PC, PS, AT, and PLG deficiencies, respectively, using the BigDye Terminator Cycle Sequencing Ready Reaction kit (Applied Biosystems, Foster City, CA, USA). When no point mutations were detected, multiple ligation-dependent probe amplification experiments were additionally performed to detect large dosage mutations.

### Statistical analyses

Categorical data are presented as number and percentage (%) and were compared using Chi-square test or Fisher exact test. Continuous variables are expressed as mean ± SD or median with interquartile range and were compared using the Student’s t test or Mann-Whitney U test, as appropriate. To adjust for confounding factors, we used a multivariate logistic regression analysis with backward stepwise method using parameters with a P value <0.10 in univariate analysis. A P-value less than 0.05 was considered statistically significant. All analyses were performed using SPSS statistical software version 23 (Chicago, IL, USA).

## Results

### Prevalence of HT

**Fig 1:**
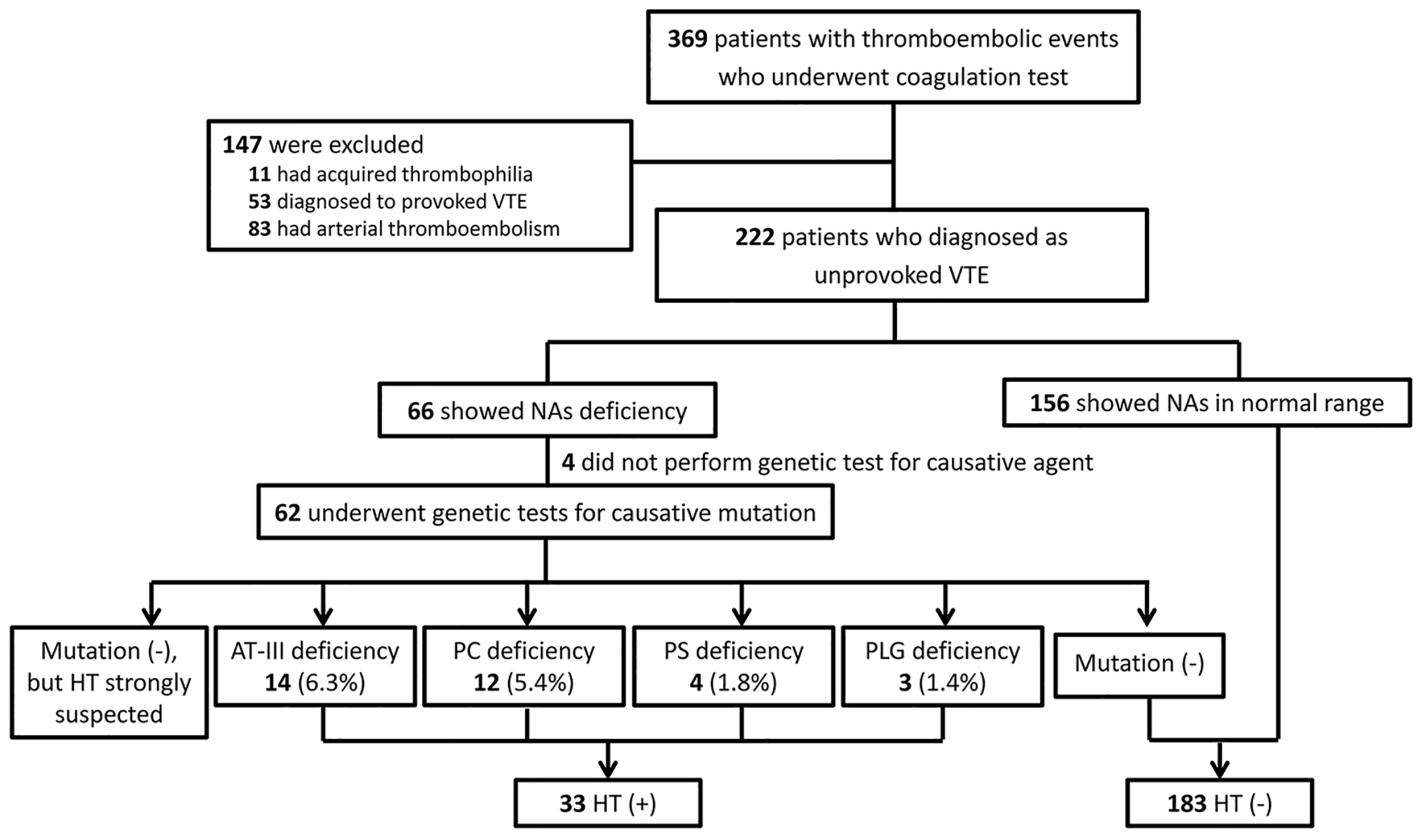
Flow diagram of the study population. VTE, venous thromboembolism; NAs, natural anticoagulants; HT, hereditary thrombophilia; AT, antithrombin III; PC, protein C, PS, protein S; PLG, plasminogen.

Among 222 patients who presented with unprovoked VTE, 66 (29.7%) had low NA level on coagulation tests, and 62 underwent genetic confirmation testing (3 did not have a follow up evaluation, and 1 declined to participate in the genetic test). Only 33 (53%) of those who showed NA deficiencies in coagulation testing were confirmed to have HT (15% of subjects with unprovoked VTE). The most common types of HT was AT III deficiency (14 of 222, 6.3%) and PC deficiency (12, 5.4%), followed by PS deficiency (4, 1.8%), and dysplasminogenemia (3, 1.4%) (Fig 1).

### Clinical characteristics of HT patients presented with unprovoked VTE

The clinical and laboratory findings of unprovoked VTE patients with or without HT are shown in Table 1. The HT group was significantly younger (37 [32–50] years vs. 52 [43–65] years, P < 0.001) and more frequently male (69.7% vs. 47.0%, P = 0.013) than the non-HT group. More than half of HT patients with unprovoked VTE had a history of previous VTE events, which is significantly higher than in the non-HT group (57.6% vs. 31.7%, P = 0.004). A family history of VTE was also more frequent in the HT group (43.8% vs. 1.9%, P < 0.001). There were no significant differences between the two groups in clinical comorbidities or laboratory findings, except level of hemoglobin (15 [13–15] vs. 13 [12–15] g/dL, P = 0.025). VTE was more frequently located in the lower extremities in patients with HT than in those without HT (87.9% vs. 67.2%, P = 0.011).

**Table 1:** Baseline characteristics of the study population.

	HT (-) N = 183	HT (+) N = 33	Total N = 216	P value
**Age, years**	52 (43–65)	37 (32–50)	50 (41–63)	< 0.001
**BMI, kg/m**^**2**^	24 (22–26)	24 (22–27)	24 (22–26)	0.459
**Males, %**	86 (47.0)	23 (69.7)	109 (50.5)	0.013
**Diabetes mellitus, %**	18 (9.8)	1 (3.0)	19 (8.8)	0.177
**Hypertension, %**	55 (30.1)	7 (21.2)	62 (28.7)	0.207
**Previous history of VTE, %**	58 (31.7)	19 (57.6)	77 (35.6)	0.004
** Age at first attack, years**	46 (37–56)	37 (27–49)	43 (35–55)	0.054
**Family history of VTE, %**	3 (1.9)	14 (43.8)	17 (8.8)	< 0.001
**Laboratory exam**				
** Hemoglobin, g/dL**	13 (12–15)	15 (13–15)	13 (12–15)	0.025
** Total bilirubin, mg/dL**	0.7 (0.4–1.0)	0.7 (0.5–1.1)	0.7 (0.4–1.0)	0.627
** AST, U/L**	23 (18–33)	22 (19–29)	23 (18–32)	0.782
** ALT, U/L**	21 (15–35)	22 (15–32)	21 (15–33)	0.755
** PT, INR**	1.1 (1.0–1.5)	1.1 (1.0–1.6)	1.1 (1.0–1.5)	0.344
** Fibrinogen, mg/dL**	307 (258–386)	292 (248–328)	303 (256–375)	0.115
** D-dimer, μg/mL**	1.0 (0.3–2.9)	0.4 (0.3–1.5)	0.8 (0.3–2.4)	0.077
**Location of VTE**				
** PE, %**	89 (48.6)	13 (39.4)	102 (47.2)	0.215
** with DVT, %**	61 (68.5)	12 (92.3)	73 (71.6)	0.066
** DVT, %**	123 (67.2)	29 (87.9)	152 (70.4)	0.011
** Lower extremity, both**	36 (29.5)	9 (31.0)	45(29.8)	0.449
** Lower extremity, proximal, %**	99 (82.5)	27 (93.1)	126 (84.6)	0.126
** PVT, %**	13 (7.1)	3 (9.1)	16 (7.4)	0.454
** SVT, %**	13 (7.1)	1 (3.0)	14 (6.5)	0.337
** CTEPH, %**	29 (15.8)	2 (6.1)	31 (14.4)	0.108
**Clinical manifestation**				
** Hypoxia**	29 (15.8)	1 (3.0)	30 (13.9)	0.034
** Shock** ^a^	11 (6.0)	0 (-)	11 (5.1)	0.154
** Tachycardia**	22 (12)	3 (9.1)	25 (11.6)	0.446

Values are median, interquartile range or n (%).

^a^ This row includes one cardiac death caused by pulmonary embolism.

ALT = Alanine aminotransferase; AST = Aspartate aminotransferase; BMI = Body mass index; CTEPH = Chronic thromboembolic pulmonary hypertension; DVT = Deep vein thrombosis; HT = Hereditary thrombophilia; VTE = Venous thromboembolism; PE = Pulmonary embolism; PT = Prothrombin time; PVT = Portal vein thrombosis; RV = Right ventricle; SVT = Splanchnic vein thrombosis.

Detailed characteristics of the HT group are given in Table 2. Patients with dysplasminogenemia tended to be older than those with other types of HT. In all types of HT, males were predominant. The first presentation of unprovoked VTE was mainly DVT rather than PE. Patients with AT-III deficiency or PC deficiency tended to have a higher previous history of VTE and family history of VTE. The details of genetic mutations are shown in Supplemental Table 1. (S1 Table)

**Table 2:** Baseline characteristics of the HT group.

	AT-III deficiency	PC deficiency	PS deficiency	Dysplasminogenemia
**Patients, %**^a^	14 (6.3)	12 (5.4)	4 (1.8)	3 (1.4)
**Age, years**	35 (33–41)	46 (32–53)	34 (32–52)	61 (44–64)
**Males, %**	8 (57.1)	9 (75.0)	4 (100)	2 (69.7)
**PE, %**	2 (14.3)	7 (58.3)	3 (75.0)	1 (39.4)
**DVT, %**	12 (85.7)	11 (91.7)	3 (75.0)	3 (100)
**Previous history of VTE, %**	9 (64.3)	9 (75.0)	0 (-)	1 (33.3)
**Family history of VTE, %**	8 (57.1)	5 (45.5)	1 (25.0)	0 (-)
**Level of NAs, % (Normal range)**	48 ± 11 (83–123)	44 ± 19 (80–161)	27 ± 17 (62–154)	60 ± 20 (75–112)

Values are n (%) or mean ± SD (median, interquartile range).

^a^ Prevalence of HT among 222 subjects diagnosed as unprovoked VTE

NAs = natural anticoagulants.

Abbreviations as in Table 1.

In multivariate analysis, age < 45 years (odds ratio [OR] 9.435, 95% confidence interval [95% CI] 2.45–36.35, P = 0.001) and a family history of VTE (OR 92.667, 95% CI 14.95–574.29, P < 0.001) were independent predictors for HT (Table 3).

**Table 3:** Results of multivariate analysis of baseline-independent predictors of hereditary thrombophilia.

Variable	OR (95% CI)	P value
Age < 45 years	9.435 (2.45–36.35)	0.001
Male	3.333 (0.85–13.04)	0.084
Previous history of VTE	2.059 (0.70–6.09)	0.192
Family history of VTE	92.667 (14.95–574.29)	<0.001

Adjusted covariates include male sex, age, previous history of VTE, family history of VTE, DVT, hemoglobin, and D-dimer.

Abbreviations as in Table 1.

### Anticoagulant therapy and recurrence of VTE

**Fig 2:**
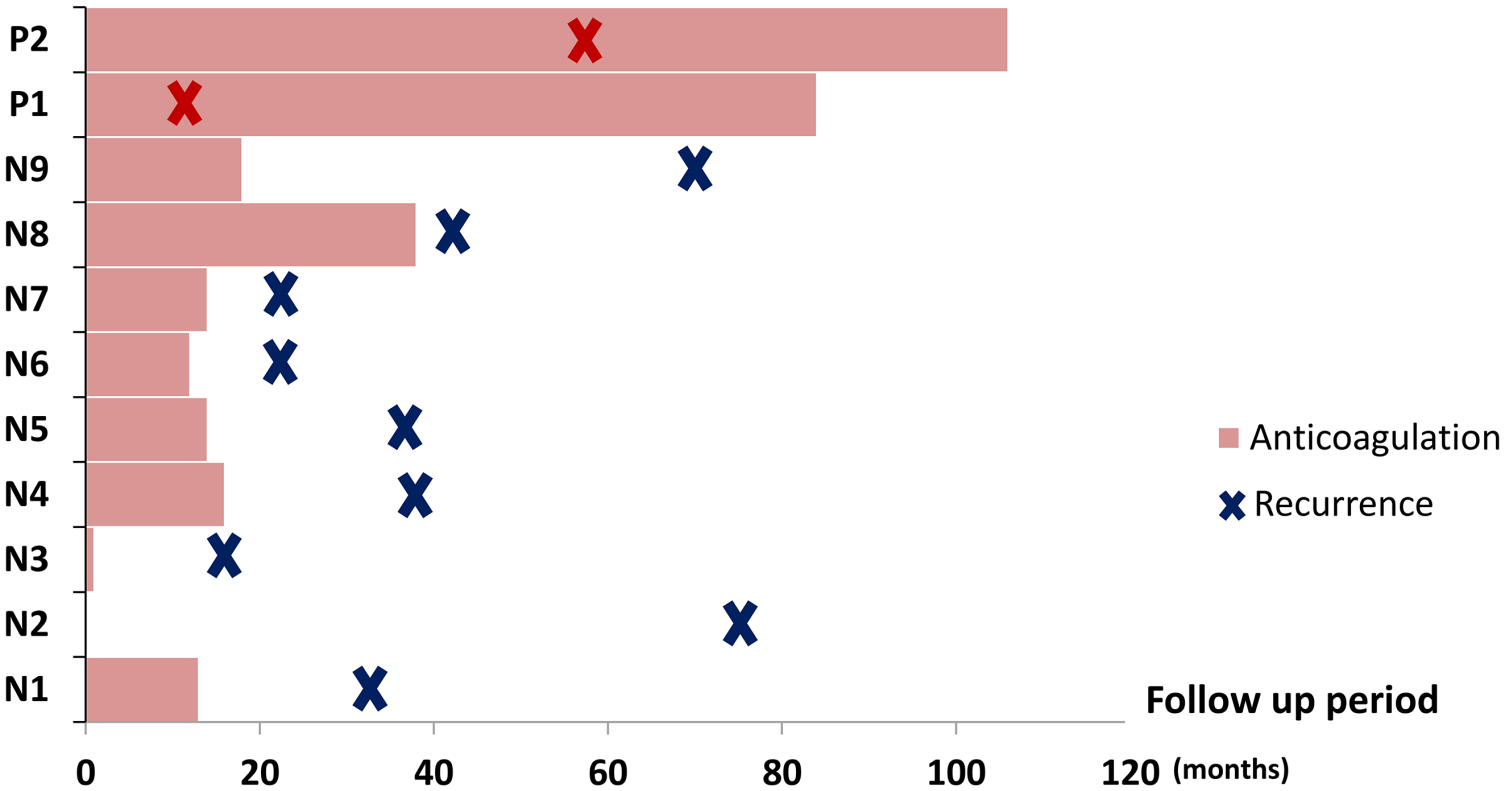
Association between recurrence of VTE and anticoagulation in patients with or without hereditary thrombophilia. N, patients without hereditary thrombophilia; P, patients with hereditary thrombophilia.

Median follow up duration was 40±38 months. Mean treatment duration of anticoagulation was much longer in the HT group than in the non-HT group (47±42 vs. 24±28 months, P < 0.001). Vitamin K antagonist (warfarin) was preferred in both groups (90.0% in HT group and 70.5% in non-HT group). Among 11 patients who experienced a recurrence of VTE, only 2 had HT (18%). About half of the recurrences were PE, followed by DVT (27%) and thrombosis in the cerebral vein, portal vein, and suprapelvic vein (9% each). Noticeably, recurrent VTE in the HT group occurred under anticoagulation, whereas all cases of recurrence in the non-HT group occurred after the end of treatment or follow-up (Fig 2).

## Discussion

In this retrospective study, we investigated the prevalence of HT and its subtypes in Korean patients with unprovoked VTE, comparing clinical manifestations and recurrences between unprovoked VTE patients with or without HT. About 15% of patients with unprovoked VTE had genetically proven HT, and AT-III deficiency and PC deficiency were most frequent subtypes. Patients with HT who developed unprovoked VTE were younger and more frequently had a family history of VTE than those without HT. However, recurrence of VTE did not differ between patients with or without HT.

The prevalence of HT varies among ethnic groups. APC resistance caused by factor V Leiden and G20210A mutations are restricted to Caucasian populations [9–11], while deficiencies of NAs have greater implications in Asian populations [11–13]. The frequency of HT has been reported heterogeneously according to the diagnostic strategy in Asian populations. Studies from other Asian countries have indicated that the prevalence of HT in VTE was 28.3% to 34% [17,18]. In Korean data, it was also reported that the prevalence of NA deficiencies with VTE was 24.3% [15]. These previous data were relied upon not the genetic abnormalities but NAs deficiency itself. Our data also showed that the prevalence of NAs deficiency in patients with unprovoked VTE was 29.7%, which was quite similar to the previous data. But, in this study, we strictly classified the patients with genetically proved HT as the HT group to investigate clinical manifestations of VTE in ‘real’ HT patients. The homogeneity of HT patients by accurate diagnosis using genetic tests resulted in those clinical manifestations being more reliable and conclusive than previous studies.

Several previous studies have considered the clinical characteristics of HT. Mateo et al. [20] reported that a family history of thrombosis and younger age (age < 45 years) were the main clinical factors that enhanced the risk of NA deficiencies. In a Japanese study of patients with DVT, the HT group was younger than the non-HT group (44.7 years vs. 52.6 years) [13]. In a recent study, Weingarz et al. [21] demonstrated an increase in the prevalence of HT with younger age (age < 40 years old) at first VTE episode, especially in cases of unprovoked VTE (OR 2.20, 95% CI 1.45–3.36, P < 0.001). In our study, the clinical features of the HT group of patients with unprovoked VTE were similar to those in previous studies. A family history of VTE and young age at presentation (<45 years) were strong predictors of unprovoked VTE caused by HT.

Generally, unprovoked VTE itself is one of the most important risk factors of recurrence, whereas the presence of NA deficiencies did not appear to increase the risk of recurrence [8,22,23]. Guidelines recommend that patients diagnosed with unprovoked VTE, regardless of HT status, extend anticoagulation beyond 3 months of therapy unless they have a high bleeding risk [24]. Interestingly, in contrast to the non-HT group, all recurrences of VTE in the HT group developed under appropriate treatment with anticoagulation in present study. Therefore, it is important to closely monitor for recurrence of VTE in patients with HT, even when they are maintaining anticoagulation.

### Study limitations

This study had some limitations. First, the study was performed retrospectively at a single center, and thus our series might not well represent the characteristics of VTE in the general population. However, our center is a tertiary referral center for thromboembolism, and we defined HT based on molecular genetic tests. Therefore, we believe that our study design had little effect on the results. Second, the test strategy of this study could have missed some recurrent mutations in Asian populations such as PC Lys192del and PS Lys196Glu, which do not result in a significant decrease of the amidolytic activity of PC and free PS Ag, respectively. [14, 25, 26].

## Conclusion

The prevalence of genetically confirmed HT in patients with unprovoked VTE was about 15%. A positive family history of VTE and young age (age < 45 years) were independent predictors for development of unprovoked VTE due to HT.

## Supporting information

S1 Data (XLSX)

S1 Table Coagulaiton and genetic test results of 33 Korean patients with hereditary thrombophilia. (DOCX)
