# Supplementary material for: The prevalence and clinical manifestation of hereditary thrombophilia in Korean patients with unprovoked venous thromboembolisms
Source: PLoS One. 2017 Oct 17;12(10):e0185785. doi: 10.1371/journal.pone.0185785 (PMC5645010; doi:10.1371/journal.pone.0185785)
Supplement: S1 Table — (DOCX) [file pone.0185785.s002.docx]

**S1 Table. Coagulaiton and genetic test results of 33 Korean patients with hereditary thrombophilia**

| **ID** | **Coagulation results** | **Gene** | **Mutation**  **(nucleotide)** | **Mutation**  **(protein)** | **Type of mutation** |
| --- | --- | --- | --- | --- | --- |
| SMC-AT-01 | AT act 58% AT Ag 98% | SERPINC1 | c.1057C>T | p.Pro353Ser | Missense |
| SMC-AT-02 | AT act 69% | SERPINC1 | c.1057C>G | p.Pro353Ala | Missense |
| SMC-AT-03 | AT act 46% | SERPINC1 | c.235C>T | p.Arg79Cys | Missense |
| SMC-AT-04 | AT act 58% | SERPINC1 | c.284A>G | p.Tyr95Cys | Missense |
| SMC-AT-05 | AT act 28% | SERPINC1 | c.243G>A | p.Trp81X | Nonsense |
| SMC-AT-06 | AT act 43% | SERPINC1 | c.992delT | p.Leu331Profs*16 | Frameshift |
| SMC-AT-07 | AT act 56% | SERPINC1 | c.490C>T | p.Arg164* | Nonsense |
| SMC-AT-08 | AT act 43% | SERPINC1 | c.[442T>C(;)  1370G>C] | p.[Ser148Pro(;)  Arg457Thr] | Missense |
| SMC-AT-09 | AT act 61% AT Ag 50% | SERPINC1 | c.243G>A | p.Trp81* | Nonsense |
| SMC-AT-10 | AT act 42% | SERPINC1 | c.550dupC | p.Leu184Profs*5 | Frameshift |
| SMC-AT-11 | AT act 56%  AT Ag 45% | SERPINC1 | c.593_594dupAT | p.Gly199Metfs*86 | Frameshift |
| SMC-AT-12 | AT act 45%  AT Ag 47% | SERPINC1 | c.1224_1227delTGAA | p.Asn408Lysfs*13 | Frameshift |
| SMC-AT-13 | AT act 53% | SERPINC1 | NM_000488.3:c.(?_-1)_(1218+1_1219-1)del |  | Large deletion |
| SMC-AT-14 | AT act 49% AT Ag 60% | SERPINC1 | NM_000488.3:c.(?_-1)_(*1_?)del |  | Large deletion |
| SMC-PC-01 | PC act 44% | PROC | c.631C>T | p.Arg211Trp | Missense |
| SMC-PC-02 | PC act 52%  PC Ag 47% | PROC | c.631C>T | p.Arg211Trp | Missense |
| SMC-PC-03 | PC act 53% | PROC | c.1218G>A | p.Met406Ile | Missense |
| SMC-PC-04 | PC act 6% | PROC | c.[629C>T  (;)631C>T] | p.[Pro210Leu(;)  Arg211Trp] | Missense |
| SMC-PC-05 | PC act 69% | PROC | c.631C>T | p.Arg211Trp | Missense |
| SMC-PC-06 | PC act 60%  PC Ag 43% | PROC | c.935C>T | p.Ser312Leu | Missense |
| SMC-PC-07 | PC act 41% | PROC | c.[565C>T(;)  574_576delAAG] | p.[Arg189Trp(;)  Lys192del] | Missense in-frame, single AA deletion |
| SMC-PC-08 | PC act 24% | PROC | c.[565C>T(;)  574_576delAAG] | p.[Arg189Trp(;)  Lys192del] | Missense in-frame, single AA deletion |
| SMC-PC-09 | PC act 24% | PROC | c.631C>T | p.Arg211Trp | Missense |
| SMC-PC-10 | PC act 53%  PC Ag 51% | PROC | c.1218G>A | p.Met406Ile | Missense |
| SMC-PC-11 | PC act 32% | PROC | c.[577_579delAAG(;)715_724delGGGGCAGTGC] | p.[Lys193del(;)  Gly239Serfs*8] | Deletion, frameshift |
| SMC-PC-12 | PC act 58% PC Ag 55% | PROC | c.1212dupG | p.Pro405Alafs*20 | Frameshift |
| SMC-PS-01 | PS Ag, f 16% | PROS1 | c.229dupG | p.Glu77Glyfs*29 | Frameshift |
| SMC-PS-02 | PS Ag, f 12% | PROS1 | c.1644G>C | p.Gln548His | Missense |
| SMC-PS-03 | PS Ag 40%  PS act 32% | PROS1 | c.1424G>T | p.Cys475Phe | Missense |
| SMC-PS-04 | PS Ag, t 44%  PS Ag, f 49%  PS act 52% | PROS1 | c.1063C>T | p.Arg355Cys | Missense |
| SMC-PLG-01 | PLG act 41% | PLG | c.1858G>A | p.Ala620Thr | Missense |
| SMC-PLG-02 | PLG act 64% | PLG | c.1858G>A | p.Ala620Thr | Missense |
| SMC-PLG-03 | PLG act 53% | PLG | c.1858G>A | p.Ala620Thr | Missense |
